# Supplementary material for: The D165H Polymorphism of QiMYB-like-1 Is Linked to Interactions between Tannin Accumulation, Herbivory and Biogeographical Determinants of Quercus ilex
Source: Int J Mol Sci. 2022 Dec 21;24(1):151. doi: 10.3390/ijms24010151 (PMC9820640; doi:10.3390/ijms24010151)
Supplement: Supplementary file 1 [file ijms-24-00151-s001.zip › ijms-2016668-supplementary.pdf]

Supplementary material

Figure S1

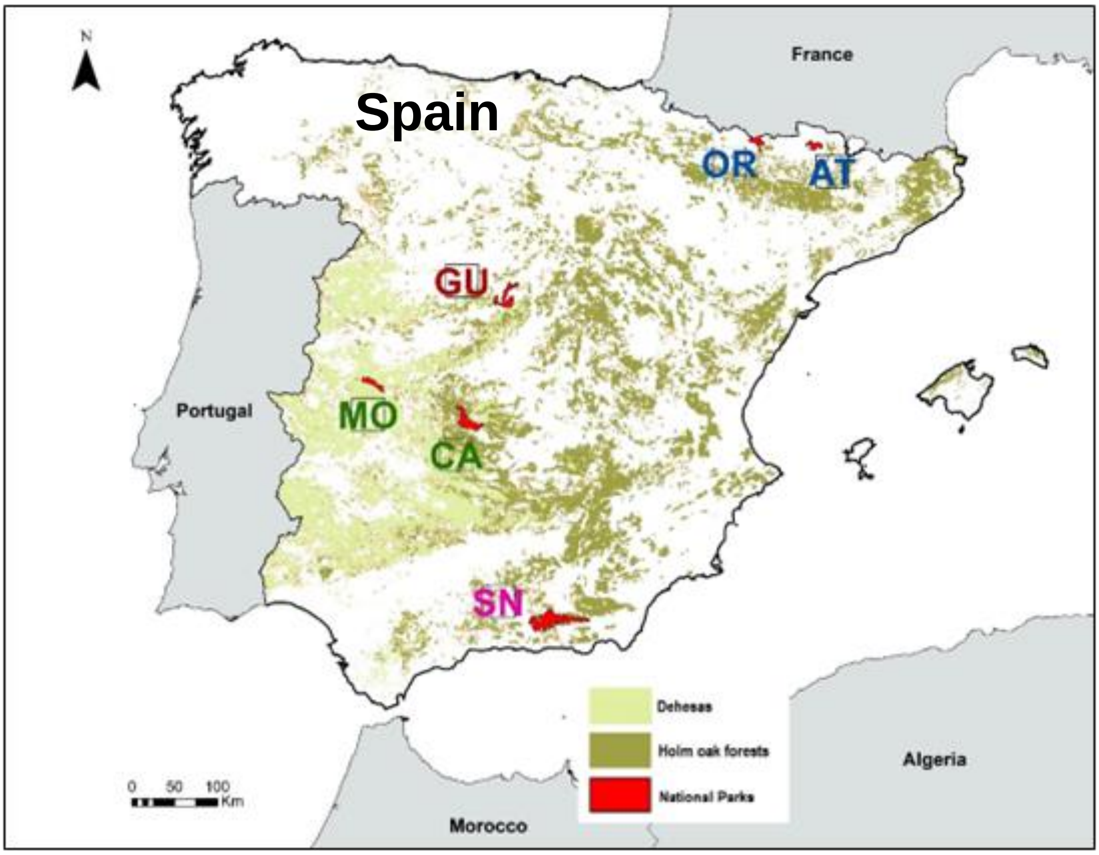

The Iberian Peninsula, with the distribution range of dehesas and holm oak forests in Spain [1, 2], and the location of the six national parks sampled in this work: Aigüestortes (AT), Cabañeros (CA) Guadarrama (GU), Monfragüe (MO), Ordesa (OR), and Sierra Nevada (SN). Parks in blue (OR+AT), Pyrenean forests; Parks in green (MO+CA), continental-Mediterranean forests.

Supplementary material

Figure S2

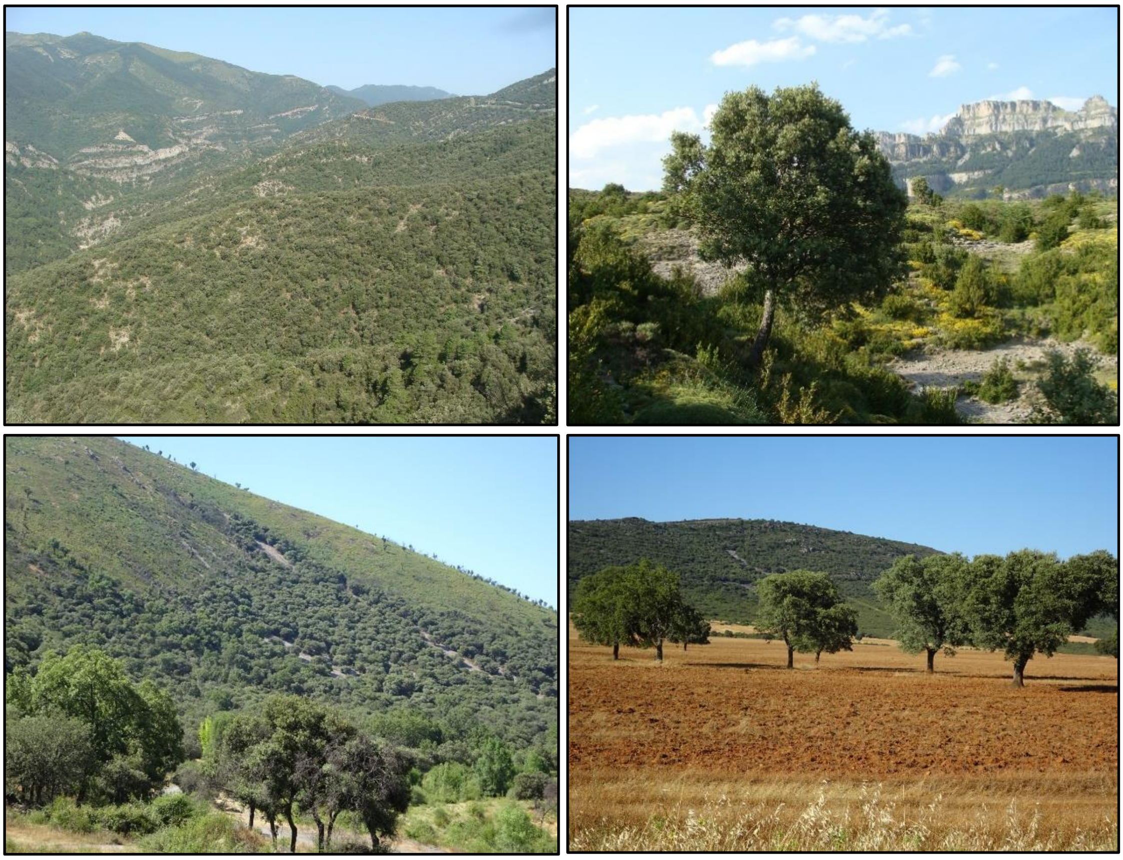

Examples of holm oak forests in the habitats sampled in this work: Ordesa National Park (upper images) and Cabañeros National Park (lower images), presenting high-density forest (images to the left) and low-density forest (images to the right).
